# The maize (*Zea mays* L.) *roothairless3* gene encodes a putative GPI-anchored, monocot-specific, COBRA-like protein that significantly affects grain yield

**DOI:** 10.1111/j.1365-313X.2008.03459.x

**Published:** 2008-04-14

**Authors:** Frank Hochholdinger, Tsui-Jung Wen, Roman Zimmermann, Patricia Chimot-Marolle, Oswaldo da Costa e Silva, Wesley Bruce, Kendall R Lamkey, Udo Wienand, Patrick S Schnable

**Affiliations:** 1 Center for Plant Molecular Biology, Department of General Genetics, Eberhard-Karls-University Tuebingen72076 Tuebingen, Germany; 2 Department of Agronomy, Iowa State UniversityAmes, IA 50011, USA; 3 Institute for General Botany and Botanical Garden, University of Hamburg22609 Hamburg, Germany; 4 Pioneer Hi-Bred International, Inc. – a DuPont CompanyJohnston, IA 50131, USA; 5 Department of Genetics, Development, and Cell Biology, Iowa State UniversityAmes, IA 50011, USA; 6 Center for Plant Genomics, Iowa State University, Ames, IA 50011-36506, USA

**Keywords:** *rth3*, COBRA-like, root hairs, maize, mutant

## Abstract

**Summary:**

The *rth3* (*roothairless 3*) mutant is specifically affected in root hair elongation. We report here the cloning of the *rth3* gene via a PCR-based strategy (amplification of insertion mutagenized sites) and demonstrate that it encodes a COBRA-like protein that displays all the structural features of a glycosylphosphatidylinositol anchor. Genes of the *COBRA* family are involved in various types of cell expansion and cell wall biosynthesis. The *rth3* gene belongs to a monocot-specific clade of the *COBRA* gene family comprising two maize and two rice genes. While the rice (*Oryza sativa*) gene *OsBC1L1* appears to be orthologous to *rth3* based on sequence similarity (86% identity at the protein level) and maize/rice synteny, the maize (*Zea mays* L.) *rth3-like* gene does not appear to be a functional homolog of *rth3* based on their distinct expression profiles. Massively parallel signature sequencing analysis detected *rth3* expression in all analyzed tissues, but at relatively low levels, with the most abundant expression in primary roots where the root hair phenotype is manifested. *In situ* hybridization experiments confine *rth3* expression to root hair-forming epidermal cells and lateral root primordia. Remarkably, in replicated field trials involving near-isogenic lines, the *rth3* mutant conferred significant losses in grain yield.

## Introduction

Root hairs enlarge the surface of the root to support the uptake of water and nutrients and the interaction with the abiotic and biotic rhizosphere (Gilroy and Jones, 2000). Unicellular root hairs are shaped by polarized growth of their tips (Kropf *et al.*, 1998), a process mediated by vesicle fusion of membranes and exocytosis of cell wall material (Wasteneys and Galway, 2003). Root hair formation can be divided into two distinct phases: bulge formation or root hair initiation and the transformation of the bulge into the tip-growing apex of the emerging root hair (Ciamporova *et al.*, 2003). Unlike alfalfa (*Medicago truncatula*), where all epidermal cells are trichoblasts which develop into root-hair-bearing cells (Sieberer and Emons, 2000), the root epidermis of most other higher plants is composed of trichoblasts and atrichoblasts which do not develop root hairs (Larkin *et al.*, 2003). In *Arabidopsis thaliana* the position of epidermal cells in a cleft between two cortical cells determines the formation of root hairs (Dolan *et al.*, 1994). In maize (*Zea mays* L.) the last division of surface cells produces two equally sized daughter cells, both of which can produce root hairs (Row and Reeder, 1957).

Despite the progress in characterizing the transcriptional networks which specify trichoblasts and atrichoblasts during epidermal patterning of the Arabidopsis root (Lee and Schiefelbein, 2002) only recently have a number of genes been identified that are involved in Arabidopsis root hair elongation. These genes have a variety of functions. The *ROOT HAIR DEFECTIVE3* (*RHD3*) gene encodes for a GTP-binding protein (Wang *et al.*, 1997) required for regulated cell enlargement, while the *TINY ROOT HAIR1* (*TRH1*) gene encodes a potassium transporter, which may be involved in the spatial localization of potassium uptake during tip growth (Rigas *et al.*, 2001). Recently, six Arabidopsis genes *MORPHOGENESIS OF ROOT HAIR 1–6* (*MRH1–6*) have been identified (Jones *et al.*, 2006) by comparing the transcriptomes of the root hair differentiation zones of the wild type and the root hair mutant *root hair defective2* (*rhd2*), which carries a null mutation in a NADPH oxidase (Foreman *et al.*, 2003). These genes encode a leucine-rich-repeat receptor-like kinase (*MRH1*), an armadillo-repeat containing kinesin-related protein (*MRH2*), an inositol-1,4,5-triphosphate 5 phosphatase-like protein (*MRH3*), a *COBRA-like 9* gene (*MRH4*), a glycerophosphoryl diester phosphodiesterase-like protein (*MRH5*), and a gene with similarity to the *Escherichia coli* universal stress protein A (*MRH6*).

In maize, only three mutants, *roothairless 1*–*3* (*rth1*, *rth2* and *rth3*), that display root hair elongation phenotypes have been reported thus far (Wen and Schnable, 1994). The only cloned monocot gene that displays a root hair elongation phenotype, *rth1*, encodes a SEC3-like protein that is a member of the putative exocyst complex which tethers exocytotic vesicles prior to their fusion (Wen *et al.*, 2005).

The *COBRA-like* gene family (Brady *et al.*, 2007; Ching *et al.*, 2006; Li *et al.*, 2003; Roudier *et al.*, 2002, 2005; Schindelman *et al.*, 2001) is divided into two subgroups that are distinguished by an N-terminal stretch of 170 amino acids (Roudier *et al.*, 2002) and contains 12 members in Arabidopsis, 11 members in rice (*Oryza sativa*) and currently nine members in the not yet completely sequenced maize genome (Brady *et al.*, 2007). Within these subgroups there are clear differences between monocot and eudicot members, including the existence of a monocot-specific clade (Brady *et al.*, 2007). Most COBRA-like proteins contain a predicted plant-specific glycosylphosphatidylinositol (GPI) anchoring site (Brady *et al.*, 2007) which is connected through an amino acid designated ω to GPI anchors (Udenfriend and Kodukula, 1995). COBRA-like proteins follow a GPI secretion path and are found in Golgi vesicles and, finally, at the outer face of the cell wall (Roudier *et al.*, 2002). Although the primary function of the *COBRA-like* gene superfamily needs to be fully determined, it appears that in general these genes are involved in various types of cell expansion and cell wall biosynthesis (Brady *et al.*, 2007).

Two major types of cell walls can be distinguished in angiosperms according to their chemical composition (Carpita and McCann, 2000). In type I cell walls, which are characteristic of dicots, cellulose and hemicellulosic xyloglucans, which are present in approximately equimolar amounts, are embedded in a pectin matrix. The type II cell walls formed in maize cells are characterized by a low pectin content, mixed link glucans or glucuronoarabinoxylans as the major hemicellulosic component and a complex network of phenylpropanoids (Carpita and McCann, 2000).

Here, we describe the cloning and characterization of the *rth3* gene encoding a COBRA-like protein that is unique to monocots and, based on the *rth3* mutant phenotype (Wen and Schnable, 1994), is required for root hair elongation and normal grain yield.

## Results

### The rth3 mutant is specifically affected in root hair elongation and grain yield

**Figure 1:**
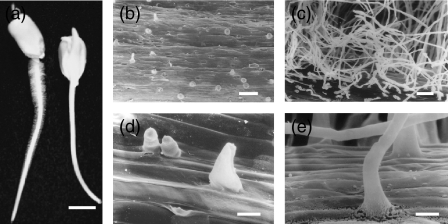
The *rth3* mutant is affected in root hair elongation. (a) Three-day-old wild-type seedling (left) and *rth3* mutant (right). (b–e) Scanning electron microscopic images of 3-day-old wild type (c, e) and *rth3* (b, d) demonstrate that root hairs are initiated in the mutant but fail to elongate. Size bars: (a) 10 mm; (b, c) 20 μm; (d, e) 200 μm.

The *rth3* mutant (Figure 1a) was previously isolated from *Mutator* transposon stocks (Wen and Schnable, 1994). In contrast to the wild type, the mutant *rth3* is affected in root hair morphology in that it initiates root hair primordia but fails to elongate them properly (Figure 1b,d; wild type, Figure 1c,e). Samples of below-ground crown roots and above-ground brace roots that were excavated from mutant plants near the time of anthesis showed no evidence of root hair elongation, confirming that the *rth3* mutation remained stable during field growth. Moreover, the *rth3* mutant did not display any apparent aberrant phenotype in the aboveground portion of the plant (including trichome formation) when grown under field or greenhouse conditions (data not shown). To assess how impaired root hairs negatively affect grain yield, three independent yield trials over 2 years were conducted on *per se* homozygous mutants versus closely related homozygous wild-type plants (see Experimental procedures). The *rth3* mutants indeed showed reductions in grain yield of 42, 37 and 19%, respectively, in the three trials (Table 1).

**Table 1:** Average yield differences between wild-type and *rth3* mutant plants

Yield	IA 97^a^	IA 94^b^	NE and KS 94^c^
Wild type (q ha^−1^)	34.03 ± 8.51	35.01 ± 12.14	44.29 ± 17.69
*rth3* (q ha^−1^)	19.63 ± 5.39	22.22 ± 12.53	35.87 ± 20.52
Yield reduction in *rth3*^d^ (%)	−42.3*	−36.5*	−19.0

a The experiment was performed in Ames and Crawfordsville, IA in 1997 and included 30 lines that were homozygous *rth3* and 30 wild-type (wt) lines.

b The experiment was performed at Ames, Ankeny, and Fairfield, IA in 1994 and included 21 lines that were homozygous *rth3* and 19 wt lines.

c The experiment was performed at North Platte, NE and Garden City, KS in 1994 and included 20 lines that were homozygous *rth3* and 20 wt lines.

d [(yield *rth3*/yield wt) − 1] × 100.

* *t*-test: *P* < 0.01.

### Mapping, cloning and sequencing of the rth3 gene

**Figure 2:**
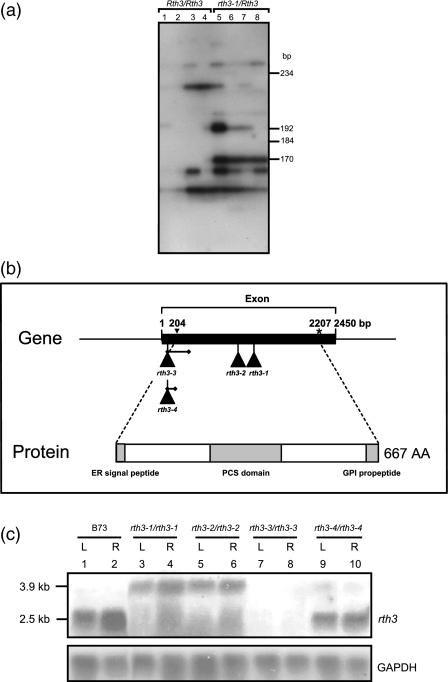
Cloning and structural features of the *rth3* gene. (a) Identification of a 170-bp DNA fragment that flanks a *Mu* transposon and that co-segregates with the mutant *rth3* phenotype in amplification of insertion mutagenized sites experiments. Lanes 1–4: genomic DNA of plants homozygous for wild–type *rth3* alleles. Lanes 5–8: genomic DNA isolated from plants heterozygous for the mutant *rth3-1* allele. (b) Structures of the *rth3* gene and RTH3 protein. Triangles indicate the positions of the *Mu* transposon insertions in the various *rth3* alleles. Horizontal bars above the triangles indicate sequences deleted adjacent to the transposon insertion sites in the *rth3-3* and *rth3-4* alleles. The start and stop codons are at positions 204 and 2207 of the cDNA (GenBank accession number AY265855). (c) Ribonucleic acid gel blot analyses of the four *rth3* mutant alleles with RNA isolated from leaves (L) and roots (R) demonstrate that *rth3-3* represents a null allele. The 3.9-kb band in alleles *rth3-1* and *rth3-2* represents *rth3* transcripts containing 1.4-kb *Mu* transposon insertions. The 2.8-kb band in allele *rth3-4* is caused by a 294-bp deletion of *rth3* sequences and the insertion of a truncated 658-bp *Mu1* transposon. The wild-type allele (B73) displays the expected 2.5-kb band.

To better understand the role of *rth3* in root hair elongation we first mapped and then cloned the gene via a PCR-based approach. Mapping of the *rth3* locus placed this gene on chromosome 1S (Wen and Schnable, 1994) between the B–A translocation breakpoints TB1-24464 (0.51) and TB1sb (0.05) in the vicinity of the *pericarp color 1* (*p1*) gene (0–3.6 cm) and the RFLP marker *ias8* (2.3 ± 1.3 cm). Cloning of the *rth3* gene was performed with the amplification of insertion mutagenized sites (AIMS) system, which allows for the detection of *Mutator* (*Mu*) transposon flanking sequences that co-segregate with the mutant phenotype (Frey *et al.*, 1998). This co-segregation analysis with genomic DNA of 126 F_1_ progeny of the cross *Rth3-1*/*Rth3-1*× *rth3-1*/*Rth3-1* revealed a 170-bp band (Figure 2a) that was amplified from each of the 69 heterozygous *rth3*/*Rth3* plants that contained the mutant allele but from none of their 57 homozygous wild-type (*Rth3*/*Rth3)* siblings. The 170-bp fragment of the mutant allele was obtained with the primer pair Mu Sel and *Hpa* II Sel/T from *Hpa* II-digested DNA (for details refer to Frey *et al.*, 1998). Screening of a seedling cDNA library using the 170-bp fragment as a hybridization probe revealed a full-length *rth3* transcript of 2450 bp (GenBank accession number AY265855), which contains an open reading frame that encodes for a 667 amino acid protein of 71.4 kDa (GenBank accession number AAQ81633). A B73 genomic clone with a size of about 10 kb, containing the complete cDNA sequence, was identified and sequenced via the Tn1000-based system (Morgan *et al.*, 1996). Comparison of the genomic and cDNA sequences established that the *rth3* gene lacks introns.

### Confirmation of rth3 cloning via independently generated alleles

We identified additional mutant alleles to confirm that the *rth3* phenotype was generated by a mutation in the gene with the GenBank accession number AY265855. Phenotypic screens for root hair defective mutants yielded three additional *rth3* alleles. Allele *rth3-2* was isolated from a screen of selfed *Mu* transposon stocks. Two additional alleles, *rth3-3* and *3-4*, were identified from a direct *Mu* transposon tagging experiment using *rth3-1* and the subsequent screening of 62 100 F_1_ progenies (see Experimental procedures). Each of these newly induced alleles displayed the same roothairless phenotype as the reference allele *rth3-1*. All four alleles contained a *Mu* transposon insertion in the transcribed region of the single exon of the *rth3* gene (Figure 2b). With reference to the *rth3* sequence deposited in GenBank (AY265855), the *Mu8* transposon inserted in allele *rth3-1* at position 1287 and is flanked by the typical direct target site duplication (TSD) of 9 bp. In the *rth3-2* allele a 9-bp TSD flanks a 1.4-kb *Mu1* transposon inserted at position 1070. Allele *rth3-3* is characterized by a 294-bp deletion from base pair 84 to 377 in which a truncated 658-bp fragment of the 5′-end of a *Mu1* transposon was inserted. A direct TSD flanking the transposon is not present in this allele. Finally, allele *rth3-4* contains a 107-bp deletion of the region defined by positions 84–190 of AY265855 in which a 1.4-kb *Mu1* transposon was inserted that was not flanked by a direct TSD.

Based on RNA samples isolated from 2-week-old leaves and roots of the wild type (B73) and the four *rth3* mutant alleles, the *rth3* gene was expressed in wild-type (B73) roots and leaves (Figure 2c). Seedlings homozygous for the *rth3-3* mutant allele did not accumulate *rth3* transcripts in either their roots or leaves. Hence, the *rth3-3* allele represents an apparently null allele. Seedlings homozygous for the *rth3-1* and *rth3-2* mutant alleles displayed a transcript of 3.9 kb when hybridized with the 170-bp *rth3* probe identified in the AIMS experiments. The 3.9-kb band is consistent with the presence of a 1.4-kb *Mu* transposon in these *rth3* alleles. The origins of the smaller hybridizing smears from these mutants are not known, but they could potentially represent multiple transcripts derived from alternative transcript initiation sites as has been observed in some other *Mu* insertion alleles (e.g. Cui *et al.*, 2003). Seedlings homozygous for the *rth3-4* mutant allele displayed a band of ∼2.8 kb, which is consistent with the deletion of 294 bp together with the insertion of a truncated 658-bp *Mu1* transposon fragment. As expected, only the 2.5-kb band was detected in wild-type (B73) seedlings.

### The rth3 gene encodes a putative GPI-anchored COBRA-like protein

Sequence similarity searches using the BlastX algorithm (Altschul *et al.*, 1997) revealed that the RTH3 protein displays significant similarity to members of the *COBRA* gene family. Consistent with the predicted COBRA relationship, the RTH3 protein contains all features of a putative GPI-anchor protein (Udenfriend and Kodukula, 1995). First, the RTH3 protein contains a hydrophilic central portion flanked by cleavable hydrophobic sequences including a N-terminal signal peptide (amino acids 1–23) for targeting the protein to the endoplasmic reticulum and a C-terminal propeptide (amino acids 649–667) required for GPI linkage. Moreover, the sequence motifs of a ω amino acid serine to which the GPI anchor is linked and the ω + 2 amino acid glycine followed by a spacer of five amino acids containing a proline and a subsequent stretch of 12 hydrophobic amino acids meet all sequence requirements of a GPI-anchored protein. Furthermore, the RTH3 protein contains a central cysteine-rich domain between amino acids 419 and 462, which includes eight cysteine residues, although the CCVS domain that appears in Arabidopsis GPI-anchored COBRA-like proteins is only conserved as CCVT. The cysteine-rich region also contained a number of predicted N-glycosylation sites which are frequently associated with GPI-anchored proteins (Roudier *et al.*, 2002). Moreover, only a weak similarity to a cellulose-binding domain II has been identified between amino acid residues 224 to 274 (*E*-value 0.009). Finally, like all members of the *COBRA* gene family, the RTH3 protein also contains a central phytochelatine synthase (PCS) domain. Phytochelatine synthetases are known to play a role in plant detoxification from heavy metals (Cobbett, 2000). However, wild type and *rth3* mutants did not display any phenotypic differences when grown in 50 and 100 μm CdCl_2_ (data not shown). In addition, expression of the RTH3 protein in the yeast *yap1* and *ycf1* mutants (Li *et al.*, 1997) grown on medium containing 50–100 μm CdCl_2_ did not complement the cadmium-sensitive phenotype of these mutants (data not shown). These results imply that there is only a structural but not a functional relationship of the RTH3 protein with known PCSs.

### The rth3 gene belongs to a monocot-specific clade of the COBRA gene family

**Figure 3:**
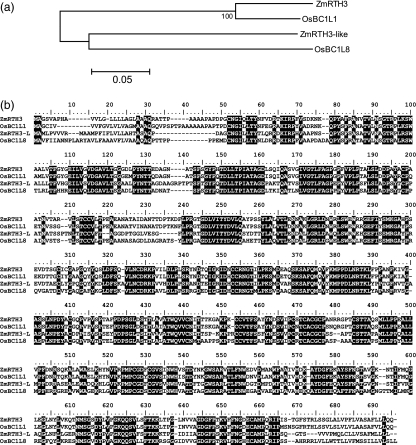
The monocot-specific clade of the N-terminal 170 amino acid residue containing subgroup of COBRA-like proteins. (a) Phylogenetic relationship of the two maize and rice genes comprising the monocot-specific clade of *COBRA-like* genes. (b) The four monocot-specific COBRA-like proteins display extensive stretches of similarity.

The *COBRA* gene family can be divided into two subgroups, which are characterized by the presence or absence of an N-terminal stretch of 170 amino acids (Brady *et al.*, 2007). The *rth3* gene belongs to a monocot-specific clade of the subgroup that contains the 170 N-terminal amino acid residues. This clade is composed of the maize genes *rth3* and *rth3-like* and the rice genes *OsBC1L1* and *OsBC1L8* (Figure 3a). All four genes contain only a single exon. Identity among the members of this clade at the protein level is between 62% and 64% (Figure 3b). Remarkably, RTH3 and OsBC1L1 share a sequence identity of 86%. Most probably the *rth3* and *OsBC1L1* genes are orthologs, not only because of their exceptional degree of sequence similarity but also because *rth3* (chromosome 1S) and *OsBC1L1* (chromosome 3) map on syntenic regions of the maize and rice genomes (Gale and Devos, 1998). The closest maize relative of *rth3*, the *rth3-like* gene, displays an overall sequence identity to RTH3 of 64% at the protein level.

### Massively parallel signature sequencing expression profiles of the rth3 gene display a variable spatial and temporal expression pattern which is distinct from that of rth3-like

**Figure 4:**
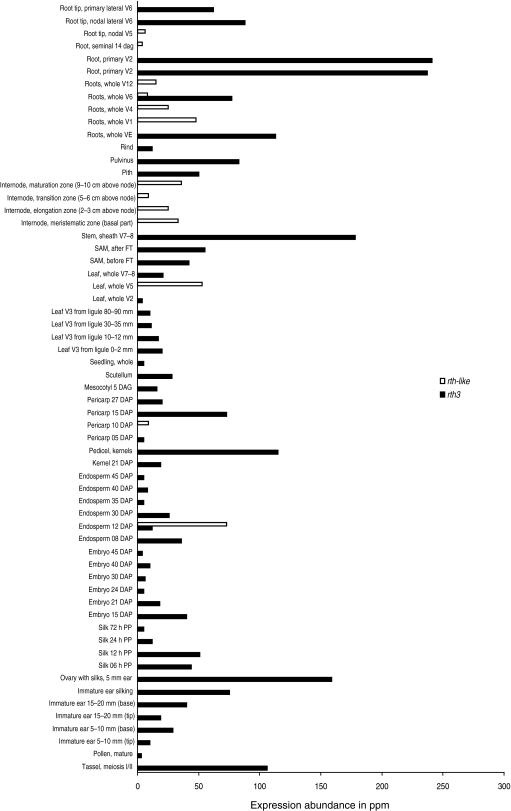
Relative expression levels (in p.p.m.) of the *rth3* and *rth3-like* genes in 50 different tissues and developmental stages from wild-type B73 inbred using the Solexa massively parallel signature sequencing system.

To obtain a detailed picture of the expression patterns of the *rth3* and *rth3-like* genes during development, DuPont's (http://www2.dupont.com/) massively parallel signature sequencing (MPSS; Brenner *et al.*, 2000) database was surveyed (Figure 4). Transcripts of *rth3* were detected in 48 different samples of the inbred line B73 representing a wide variety of tissues and developmental stages of the maize plant. The young primary root (V2 stage), which was represented by two independent libraries, displayed the highest *rth3* transcript abundance (about 240 p.p.m.) consistent with the observed root hair phenotype of the *rth3* mutant. Within reproductive tissues, *rth3* exhibited its highest expression in ‘ovaries with silk’ of young 5-mm ears (160 p.p.m.). Lack of expression in this tissue in the mutant might partially explain the significantly reduced grain yield of the *rth3* mutant. In contrast to *rth3*, the *rth3-like* gene is expressed in only a limited number of tissues including different stages of root development and all zones of the internode. Moreover, *rth3* transcript accumulates in 12-days after pollination (DAP) endosperm, 10-DAP pericarp and V5 leaves. No expression of the *rth3-like* gene was detected in reproductive tissue and embryos. Hence the closely related genes *rth3* and *rth3-like* display complementary expression patterns. While expression in the internode was only detected in *rth3-like*, both genes exhibit a mutually exclusive expression pattern in distinct phases of root, pericarp and leaf development except for 12-DAP endosperm where both *rth3* and *rth3-like* are expressed.

### In situ hybridization experiments demonstrate rth3 expression in trichoblasts and lateral root primordia in the differentiation zone of the primary root

**Figure 5:**
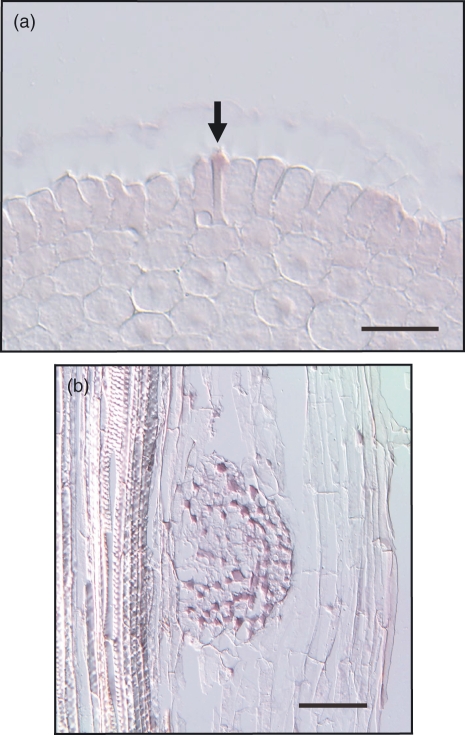
*In situ* localization of *rth3* transcripts in the differentiation zone of maize primary roots reveal expression in distinct epidermal cells (a) and in emerging lateral root primordia (b). Size bars: (a) 50 μm; (b) 100 μm.

The MPSS analyses demonstrated that *rth3* displays its strongest expression in young primary roots where the *rth3* root hair phenotype is manifested. Hence, we performed *in situ* hybridization experiments to obtain a detailed understanding of the spatial expression patterns of *rth3* in the root hair zone of the primary root. Consistent with the mutant phenotype, *rth3* transcripts were detected in the apical tips of elongating, root-hair-forming, epidermal cells (Figure 5a). Additionally, *rth3* was also expressed in young lateral root primordia, which are not associated with a mutant phenotype in *rth3* (Figure 5b).

## Discussion

In recent years a number of maize mutants affected in various aspects of root development have been identified (summarized in Hochholdinger *et al.*, 2004a, Hochholdinger *et al.*, 2004b). However, only two of the genes impaired in these mutants have been cloned (Taramino *et al.*, 2007; Wen *et al.*, 2005). Among these, the *rth1* gene, which like *rth3* displays a root hair phenotype, encodes a *sec3* homolog involved in exocytotic vesicle fusion (Wen *et al.*, 2005). While the *rth3* mutant is specifically affected in root hair elongation, the *rth1* mutant displays pleiotropic effects during plant development (Wen and Schnable, 1994).

The *rth3* gene encodes a 71.4 kDa protein that displays significant similarity with proteins of the *COBRA-like* gene superfamily. A predicted GPI anchor is present in all maize *COBRA-like* genes (Brady *et al.*, 2007), but was predicted for only 9 of 12 *COBRA-like* genes from Arabidopsis and for only 9 of 11 rice *COBRA-like* genes. Another characteristic feature of COBRA-like proteins is the presence of a central PCS domain (Figure 2b). Phytochelatines play a critical role in cadmium detoxification of plants by sequestering phytochelatine–cadmium complexes into vacuoles (Cobbett, 2000). Functional tests for PCS activity of the *rth3* gene by complementation of the yeast cadmium-sensitive mutants *ycf1* and *yap1* (Li *et al.*, 1997) were negative, and the growth behavior of wild-type versus mutant seedlings in cadmium solution also did not display any differences (data not shown) that could be related to a function of RTH3 in cadmium detoxification. This demonstrates that *rth3* does not contain a functional PCS domain. In addition, phylogenetically, functional PCS proteins are only distantly related to COBRA-like proteins. The complementation of a yeast mutant deficient in a PCS by Arabidospsis COBRA (Leuchter *et al.*, 1998) was attributed to the cysteine-rich region of the protein that could bind metal ions (Roudier *et al.*, 2002), not to the PCS domain of the COBRA protein.

COBRA-like proteins can be divided into two subgroups based on the presence of an N-terminal stretch of 170 amino acid residues (Roudier *et al.*, 2002). The RTH3 protein contains this additional N-terminal domain. Within these subgroups there are significant differences between monocot and dicot COBRA-like proteins. These differences complicate efforts to identify orthologous genes between Arabidopsis and rice or maize (Brady *et al.*, 2007). On the other hand, for most maize genes a likely rice orthologous counterpart could be assigned based on phylogenetic reconstructions (Brady *et al.*, 2007). The *rth3* gene belongs to a monocot-specific clade composed of two maize and two rice genes. The *rth3* gene is the first member of this monospecific clade that has been characterized based on a specific mutant phenotype. Most likely *OsBC1L1* is the rice homolog of *rth3*, not only because of the high degree of identity at the protein level (86%) but also because the two genes map to syntenic regions of the maize and rice genomes. On the other hand, the closest maize relative, *rth3-like* appears not to be a functional homolog of *rth3* despite the structural similarity on the protein sequence level. The MPSS expression data revealed expression of *rth3* in 48 different tissue types. The *rth3-like* gene, however, was only expressed in one of these tissues (14-DAP endosperm) but in addition was also transcribed at different stages of root and internode development.

Thus far, only a few of the Arabidopsis, rice and maize *COBRA-like* genes have been functionally characterized based on their mutant phenotypes. Within the subgroup that lacks the N-terminal stretch of 170 amino acids the *AtCOBRA* gene is required for the oriented deposition of cellulose microfibrils during anisotropic expansion of most organs during post-embryonic development (Roudier *et al.*, 2005). Moreover, the *AtCOBL4* gene that is one of the few COBRA-like proteins without a GPI anchor that is required for cellulose biosynthesis in the secondary cell wall (Brown *et al.*, 2005; Persson *et al.*, 2005). In monocots, the rice *brittle culm 1 like* (Li *et al.*, 2003) and the maize gene *brittle stalk 2* (Ching *et al.*, 2006) genes affect the mechanical strength of the plants. These genes belong to the same clade within the subgroup that lacks the 170-amino-acid N-terminus and are most likely orthologs. Moreover, they are currently the only example for which an Arabidopsis ortholog (*AtCOBL4*) has been postulated (Brady *et al.*, 2007).

In the second subgroup whose members are defined by the presence of the N-terminal stretch of 170 amino acids, only *AtCOBL9* is currently associated with a mutant phenotype. Arabidopsis *cobl9* mutant root hairs are shorter and wider than wild-type root hairs and burst soon after the establishment of tip growth at an unpredictable point of the root hair surface (Jones *et al.*, 2006). Although the *rth3* mutant also displays a root hair phenotype, its phenotype differs from that of Arabidopsis *cobl9* mutants. Unlike Arabidopsis *cobl9* mutants, the root hairs of *rth3* seedlings do not burst, but instead cease elongation soon after initiation. The *rth3* gene belongs to a different, and monocot-specific, clade of subgroup II than the Arabidopsis *cobl9 gene*. Moreover, only a relatively low degree of sequence similarity (namely 49%) exists between AtCOBL9 and RTH3 on the protein level. In contrast, the confirmed orthologs AtCOBL4 versus ZmBK2 and OsBC1 exhibited sequence identities between the monocot and dicot members of between 72% and 77%, respectively. Finally, according to MPSS data, the *rth3* gene is expressed in almost all tissues, with highest expression in young primary roots. In contrast, a RT-PCR analysis of *AtCOBL9* did not detect expression in 2- to 7-week-old roots and leaves but only in 7-week-old flowers (Roudier *et al.*, 2002), and this only after 30–40 cycles of PCR. This difference in gene expression is also confirmed by more detailed microarray data of *AtCOBL9* (Brady *et al.*, 2007) which again indicate very low expression of this gene in the radicle and roots as compared to other organs. This discrepancy between the sequence and gene expression level might imply that *rth3* and *AtCOBL9* are not orthologous although both genes display root hair phenotypes. The functional differences between these genes might be related to the different composition of Arabidopsis (class I) and maize (class II) cell walls (Carpita and McCann, 2000) and hence alternative functions of these cell wall proteins in the distinct cell wall context.

Despite the specific root hair phenotype of the *rth3* mutant, the gene is expressed in all analyzed wild-type tissues and all studied developmental stages between embryogenesis and post-embryonic vegetative and generative development. Two peaks of expression might be associated with the observed phenotype. First, the highest expression of *rth3* was observed in young (V2) primary roots. Such young roots bear a high concentration of root hairs. Lack of *rth3* transcripts in these roots might therefore be directly associated with the observed root hair phenotype. During generative development the highest expression was observed in ovaries with silks from young 5-mm ears. Again this expression pattern might be directly associated with the reduced yield that is observed in *rth3* mutant plants. However, reduced yield might also alternatively be indirectly associated with the reduced nutrient transport in the plant due to the significant reduction of the absorbing surface of the root hair, which provides up to 77% of the root surface in crops (Parker *et al.*, 2000). Normal *rth3* gene function may require expression beyond a certain threshold level that is only surpassed in young primary roots and perhaps in young ovaries, which might explain the reduced yield of *rth3* mutants.

*In situ* hybridization experiments demonstrated that *rth3* is expressed in epidermal cells that develop into root hair. This is in line with the observed phenotype of the *rth3* mutant which does not allow proper elongation of root hairs in the absence of the *rth3* gene product. Remarkably, *rth3* expression was also detected in lateral root primordia, although no lateral root defects were observed in *rth3* mutants. This implies that *rth3* might also play a role in the complex molecular networks that are involved in lateral root formation but that the *rth3* function might be redundant in lateral root formation. Although the *rth3* gene is expressed in all analyzed tissues and developmental stages, based on the very specific phenotype of the *rth3* mutant and the lack of a significant cellulose-binding domain it is unlikely that RTH3 is a general regulator of cell expansion and cell wall biosynthesis. More likely, the *rth3* gene plays a specific role in the tightly coordinated network of genes responsible for root hair elongation in the epidermis while the spatial and temporal expression of other *COBRA-like* genes might be required for the regulation of cell expansion in other plant organs. Future identification of the interaction partners of RTH3 will enhance our understanding of the molecular networks involved in root hair elongation in monocots.

## Experimental procedures

### Isolation of new rth3 alleles and maintenance of the mutant stocks

The *rth3* reference allele *rth3-1* described in Wen and Schnable (1994) was maintained by backcrossing heterozygous plants to the inbred line B73 over numerous generations as described previously (Wen and Schnable, 1994). An additional allele, *rth3-2*, was identified by a forward genetic screening of *Mutator* stocks at Pioneer Hi-Bred. The alleles *rth3-3 and rth3-4* were obtained from the 62 100 progeny of a directly tagged population using *rth3-1*/*rth3-1* as males crossed to females from highly active *Mutator* stocks (*Rth3*/*Rth3 Mu* X *rth3-1*/*rth3-1*).

### Genetic mapping of the rth3 gene

After the initial B–A translocation mapping of *rth3* to the short arm of chromosome 1 (Wen and Schnable, 1994), a higher-resolution mapping experiment was conducted using phenotypic markers. A stock carrying the phenotypic markers, *sr1* (*striate 1*), which displays a pale green leaf phenotype, and *P1-rr* (*pericarp color1*) which regulates the synthesis of a red phlobaphene pigment in maize floral organs, with suffix *rr* indicating red pericarp and cob color, was crossed to *rth3-1*/*rth3-1* plants with green leaves and a white pericarp and cob (*P1-ww*) (*sr1 Rth3 P1-rr*/*sr1 Rth3 P1-ww*× *Sr1 rth3-1 P1-ww*/*Sr1 rth3-1 P1-ww*). Progenies of this cross were backcrossed to both parent lines (*sr1 Rth3 P1-rr* (or *P1-ww*)/*Sr1 rth3-1 P1-ww* × *Sr1 rth3-1 P1-ww*/*Sr1 rth3-1 P1-ww* and *sr1 Rth3 P1-rr* (or *P1-ww*)/*Sr1 rth3-1 P1-ww*× *sr1 Rth3 P1-rr*/*sr1 Rth3 P1-ww*). The pale-green-leaf phenotype of *sr1* was scored for each progeny of these crosses 1 month after germination in the field and the *rth3* and *p1* genotypes were identified from scoring the root hair phenotypes and the pigmentation of the pericarp and cob of their selfed-pollinated ears, respectively (Coe, 1994). In addition, the molecular markers *npi286* and *ias8* were used as to genotype Ben Burr's recombinant inbred lines (Burr and Burr, 1991; Burr *et al.*, 1988).

### The AIMS system to clone rth3

The *rth3* gene was cloned using the PCR-based AIMS system as described in Frey *et al.* (1998). Homo- or heterozygosity of the analyzed plants was confirmed by selfing and subsequent segregation analysis of the progeny.

### RNA gel blot analyses

Ribonucleic acid was isolated from 2-week-old leaves and roots by a phenol/SDS method (Ausubel *et al.*, 1994). An oligo (dT)-cellulose column (Molecular Research Center, http://www.mrcgene.com/) was used to purify mRNA. Ten micrograms of mRNA per sample was subjected to electrophoresis and transferred to nylon membranes. Transfer, crosslinking, probe labeling, pre-hybridization, hybridization and post-hybridization washes of RNA gel blots were conducted as previously described (Stinard *et al.*, 1993).

### Survey of the massively parallel signature sequencing database

The Solexa MPSS technology (Brenner *et al.*, 2000) allows for the quantification of 17-bp signature sequences beginning with the nucleotides GATC in populations of 2 × 10^5^ to 2 × 10^6^ cDNAs of a defined developmental stage of an organ. These 17-bp signature sequences almost always correspond to unique cDNAs by direct sequence matching, thus allowing for the quantification of the abundance of a particular cDNA in a sample representing a particular organ and developmental stage. The MPSS data were normalized and filtered according to the Solexa protocol.

### In situ hybridization experiments

Five-day-old primary root samples were fixed at 4°C overnight in 4% formaldehyde in phosphate-buffered saline, dehydrated in an ethanol series and embedded in paraffin wax (Paraplast Plus; McCormick, http://www.mccormickscientific.com/). Embedded roots were sectioned using a Leica RM 2035 rotary microtome (http://www.leica.com/) and mounted on Super-Frost Plus slides (Menzel GmbH; http://www.mendel.de/). The template for the antisense *rth3 in situ* probe was amplified with primers RTH3F_as (5′-ACATGCGCGGGCCCCACTTTACAAGCG-3′) and RTH3RT7 (5′-GGGGGGTAATACGACTCACTATAGGGGTCGTCGCCCTCT-GCGC-3′) and corresponds to positions 19 to 668 of GenBank accession AY265855 followed by a T7 promoter site. The template for the sense probe was amplified accordingly using primers RTH3FT7 (5′-GGGGGGTAATACGACTCACTATAGGG-CTCTCCTCGGCACATGC-3′) and RTH3R_se (5′-CAGCTGGATGCCGTTGCAGCC-3′) with the T7 promoter preceding the *rth3* sequence. Digoxigenin-labeled RNA probes were transcribed using T7 RNA polymerase (Roche, http://www.roche.com/) according to the manufacturer's instructions. The RNA *in situ* hybridizations were performed according to Jackson (1992). Nomarski images were taken using a Zeiss Axioplan 2 microscope (http://www.zeiss.com/) in combination with a Photometrics Cool Snap camera (Roper Scientific GmbH, http://www.roperscientific.com/).

### Sequence analysis and phylogeny

Glycosylphosphatidylinositol-anchor prediction was performed with big-PI Predictor software (http://mendel.imp.ac.at/gpi/gpi_server.html; Eisenhaber *et al.*, 1999). The signal peptide was predicted with WoLF PSORT software (http://wolfpsort.org/; Horton *et al.*, 2006). The hydropathy plot according to Kyte and Doolittle (1982) was generated with ProtScale software (http://expasy.org/tools/protscale.html; Gasteiger *et al.*, 2005). Cellulose-binding domain analysis was performed with Superfamily 1.69 software (http://supfam.mrc-lmb.cam.ac.uk/SUPERFAMILY/hmm.html; Gough *et al.*, 2001). Prediction of N-glycosylation sites was performed via the NetNGlyc 1.0 server (http://www.cbs.dtu.dk/services/NetNGlyc/). Phylogenetic analysis of the monocot-specific clade of COBRA-like proteins was performed as described in Zimmermann and Werr (2005). Pairwise alignment of the monocot-specific COBRA-like proteins for evaluation of sequence identity was performed with the MegAlign algorithm of the DNAstar software package (DNAstar Inc., http://www.dnastar.com/) via the Lipman–Pearson method.

### Grain yield tests

Grain yield (quintal/hectare) of wild type was compared to that of the *rth3* mutant in three different experiments in Iowa, Kansas and Nebraska over 2 years (1994 and 1997). Yield plots were planted at a density of approximately 69 000 plants ha^−1^ in two rows per plot in a split-plot complete random design and replicated at least three times at all locations. The experiments were conducted with no visible water stress, and pests and weeds were adequately controlled throughout the season. Selfed progeny from *rth3-1*/*Rth3* heterozygotes were again self-pollinated. The root hair phenotypes of approximately 15 kernels from each twice selfed family were examined as described (Wen and Schnable, 1994). Families that did not segregate for root hair mutants (*Rth3*/*Rth3*) and those that did not segregate for non-mutants (*rth3-1*/*rth3-1*) were separately pooled to comprise the mutant and wild-type controls used in the yield tests. To ensure stability of the mutant phenotype in field-grown plants, the root clumps and brace root samples from several randomly chosen mutant and wild-type plants near flowering time were excavated from several observation plots and gently washed with tap water. Small sections of above-ground brace roots, below-ground primary and lateral roots were placed in sterile water in Petri dishes and examined stereoscopically for evidence of root hairs.
